# Non-conversion of sputum culture among patients with smear positive pulmonary tuberculosis in Cameroon: a prospective cohort study

**DOI:** 10.1186/1471-2334-14-138

**Published:** 2014-03-11

**Authors:** Eric Walter Pefura-Yone, André Pascal Kengne, Christopher Kuaban

**Affiliations:** 1 Department of Internal Medicine and Subspecialties, Faculty of Medicine and Biomedical Sciences, University of Yaounde I, Yaounde, Cameroon; 2 Pneumology service, Yaounde Jamot Hospital, Yaounde, Cameroon; 3 South African Medical Research Council & University of Cape Town, Cape Town, South Africa; 4 Faculty of Health Sciences, University of Bamenda, Bamenda, Cameroon

**Keywords:** Mycobacterium tuberculosis, Culture conversion, Outcome, Cameroon

## Abstract

**Background:**

We investigated the determinants of sputum culture non-conversion following intensive phase of treatment, and assessed the effects on the outcome among patients treated for a first episode of smear positive tuberculosis (TB).

**Methods:**

This was a prospective cohort study spanning October 2009 to May 2012, among patients treated for a first episode of smear positive pulmonary tuberculosis in the Chest service of the Yaounde Jamot Hospital, Cameroon. Logistic regressions models were used to relate baseline characteristics with non-conversion of sputum cultures after the intensive phase of treatment.

**Results:**

A total of 953 patients were admitted to the service during the study period, including 97 (10.2%) who had a positive sputum smear at the end of the intensive phase of anti-tuberculosis treatment. Eighty-six patients with persistent of smear positive sputa at the end of intensive phase of TB treatment were included, among whom 46 (53%) had positive sputum culture for *Mycobacterium tuberculosis* (C+). The absence of haemoptysis [adjusted odd ratio 4.65 (95% confidence intervals: 1.14-18.95)] and current smoking [7.26 (1.59-33.23)] were the main determinants of sputum culture non-conversion. Of the 46C + patients, 7 (15%) were resistant to at least one anti-tuberculosis drug. Treatment failure rate was 28% among C + patients and 8% among C– patients (p = 0.023). The sensitivity and specificity were 78.6% and 55.4% for culture non-conversion after intensive treatment, in predicting anti-TB treatment failure.

**Conclusions:**

Failure rate is high among patients with positive sputum culture after intensive treatment, even in the absence of multi-drug resistant bacilli. Treatment should be closely monitored in these patients and susceptibility to anti-tuberculosis drugs tested in the presence of persistent positive smears following the intensive phase of treatment.

## Background

Tuberculosis (TB), despite the existing prevention and control strategies remains a major public health problem. In the year 2011 alone for example, 8.7 million new cases of the disease were declared around the world, with a large proportion of these occurring in developing countries [1]. In developing countries, insufficient resources are a deterrent to routine testing of the susceptibility of *Mycobacterium tuberculosis* (*M. tuberculosis*) to antituberculosis drugs prior to the treatment. In this context, direct sputum examination for the presence of acid-fast bacilli (AFB) is routinely used both to assess the contagiosity and to predict the outcome of care during the treatment of patients with smear positive pulmonary tuberculosis (SPPTB) [2,3]. A negative sputum smear on direct sputum examination at the end of the intensive phase of treatment is equally used as criteria for the transition to the continuation phase [2,3]. About 5 to 30% of patients with a first episode of SPPTB according to some reports remain positive after two months of treatment. A variable proportion of these patients still have viable *M. tuberculosis* and as such continue to contribute to the spread of the disease [4]. It also appears that the course of the disease in such patients is less favourable with high rates of treatment failure and treatment discontinuation [5,6].

The relationship of positive smear with culture conversion for *M. tuberculosis* at the completion of the intensive phase of antituberculosis treatment as well as its impact if any, on the outcome of care have in general have been less investigated. In a study by Su et al. [7] the non-use of directly observed treatment (DOT) and resistance to rifampicin were reported to be the main factors associated with non-conversion of sputum culture at the end of intensive phase of antituberculosis treatment in Taiwan. The aim of this study was to determine the frequency and identify factors associated with non-conversion of sputum culture for *M. tuberculosis* as well as assess its impact on the outcome of patients treated for a first episode of SPPTB with persistent sputum smear positivity at the end of the two month intensive phase of antituberculosis treatment.

## Methods

### Study setting

The study was conducted in Unit A of the Chest service of the Yaounde Jamot Hospital (YJH), the reference hospital for respiratory diseases for the capital city of Cameroon (Yaounde) and surrounding areas. In this service, the diagnosis of SPPTB is made on the basis of a suggestive clinical history and the presence of AFB on at least one of two sputum samples submitted on two consecutive days by the patient for microscopic examination, after staining by the auramine technique [8]. Cultures for mycobacteria are hardly ever done because of costs.

All patients with a first episode of SPPTB are hospitalized during the intensive phase of treatment which lasts for two months. The total duration of treatment is six months. It consists of a two month intensive phase of daily rifampicin (R), isoniazid (H), pyrazinamide (Z) and ethambutol (E), followed by a four month continuation phase of daily R and H on outpatient basis. Treatment in the intensive phase is administered under direct supervision of the health personnel while compliance to treatment during the continuation phase is assessed by monthly return for drug collection.

During the six month treatment period, the patients’ sputum is checked thrice for the presence or absence of AFB by direct microscopic examination. This is done at the end of the two month intensive phase and at the end of fifth and sixth month of treatment. In patients whose sputum smears remain positive at the end of the two month intensive phase of treatment an additional month of intensive phase treatment is given followed by the continuation phase. The outcomes of patients at the end of the six months of treatment is recorded into one of the following six mutually exclusive categories according to the recommendations of the Union and Wold Health Organisation [1,8] 1) cured: treatment completed with a negative sputum smear in the last month of treatment and on at least one previous occasion; 2) treatment completed: patient who has completed treatment but does not meet the criteria to be classified as a cure or failure; 3) treatment failure: patient who is sputum smear positive at five months or later during treatment; 4) died: patient who dies for any reason during the course of treatment; 5) defaulted: patient whose treatment is interrupted for two consecutive months or more; 6) transferred out: patient who has been transferred to another recording and reporting unit and for whom treatment outcome is unknown. Patients are considered as having treatment success if they fall in categories (1) and (2).

### Methods

A prospective cohort of all patients aged 15 years and above, hospitalised in the service between October 2009 and May 2012 (32 months duration) with a first episode of SPPTB and whose sputum smears remained positive for AFB at the end of the two month intensive phase of treatment was studied. After obtaining informed consent, each patient’s medical records were reviewed. The following baseline information on admission was extracted from each patient’s file: age, sex, duration of illness before admission, clinical signs, alcohol and tobacco use, body mass index, HIV serostatus as well as the CD4 cell count for those with HIV infection. The result of the sputum smear of each patient with a higher bacillary load of the two examined for diagnosis as well as that of the sputum smear examined at the end of the two month intensive phase of treatment were also recorded. Bacillary load was measured by the semi-quantitative method in use in tuberculosis control programmes (i.e. 1+, 2+, 3+) [8]. An admission chest X-ray for each patient was reviewed and interpreted. Each chest radiograph was divided into six zones by eye and the presence or absence of parenchymal disease was recorded for each zone. The presence or absence of cavities was also noted.

A sputum sample was collected from each patient with persistent positive sputum smears after two months of treatment for culture and drug susceptibility testing to the different antituberculosis drugs. Cultures for *M. tuberculosis* were carried out using the classical Lowenstein-Jensen (LJ) solid medium and susceptibility to different antituberculosis drugs (rifampicin, isoniazid, ethambutol and streptomycin) was based on the standard proportion method. Sputum specimens collected from each eligible patient were decontaminated and inoculated onto LJ. Cultures were incubated at 37°C and read weekly for growth for a maximum duration of 8 weeks. The identification of the cultured strains was based on the following: culture aspects on the two media, resistance to thiophene-2-carboxylic acid hydrazide (TCH, 2 mg/l), susceptibility to para-amino-salicylic acid (PAS, 0.5 mg/l), reduction of nitrates, niacin production and catalase production at 22°C and 68°C. Drug susceptibility testing of *M. tuberculosis* complex strains isolated from cultures was performed using the indirect proportion method on LJ medium as described by Canetti et al. [9]. A patient was considered to harbour drug-resistant M. tuberculosis if the number of colonies growing on a medium containing 0.2 mg/l INH, 2 mg/l EMB, 4 mg/l SM or 40 mg/l RMP exceeded 1% of the growth on a drug-free culture plate.

All the patients with positive sputum smears at the end of the initial intensive phase of treatment were given an additional month of intensive phase therapy followed by a three month continuation phase of treatment. Sputum smears of each patient were again examined for AFB at the end of the fifth and sixth month of treatment. Outcomes of treatment were then recorded accordingly at the end of six months of therapy. The study was approved by the administrative authorities of the YJH and Cameroon National Ethics Committee.

### Statistical analysis

Data were analysed with the use of the SPSS® statistical software version 17 (SPSS Inc., Chicago, USA). Chi square and Fisher’s exact tests were used to compare proportions, and Mann–Whitney *U* test was used to compare quantitative variables in both groups. Logistic regression analysis was used to determine the baseline factors associated with culture non-conversion at the end of the second month of intensive phase of antituberculosis therapy. Potential candidate predictors were first investigated in univariate analysis and significant variables (based on a threshold probability of 0.1) were all entered in the same multivariable logistic model to determine independent predictors for culture non-conversion in patients with persistent sputum smear positivity after two months of intensive therapy. A p-value <0.05 was used to characterize significant results.

## Results

### Study population

A total of 953 patients with SPPTB were admitted in the service over the study period. Ninety-seven (10.2%) of these patients presented persistent sputum smear positivity at the end of the two month intensive phase of antituberculosis therapy. Nine of the 97 patients did not have a sputum culture for *M. tuberculosis* and two patients’ cultures yielded atypical mycobacteria. They were therefore excluded from further analysis. Of the 86 patients analysed, 63 (73.3%) were men and their median (25^th^ – 75^th^ percentiles) age was 33 (26–42) years (Table 1). Forty-six (53.5%) of the 86 patients had positive culture for *M. tuberculosis.*

**Table 1:** **Clinical and radiological characteristics of patients with positive sputum smear at the end of the second month of intensive treatment, with positive (C+) or negative (C–) culture for *Mycobacterium tuberculosis***

**Characteristics**	**Overall (n = 86)**	**C + (n = 46)**	**C– (n = 40)**	**p**
Sex				
Men, n (%)	63 (73.3)	35 (76.1)	28 (70)	0.525
Women, n (%)	23 (26.7)	11 (23.9)	12 (30)	
Median age, years (25^th^-75^th^ percentiles)	33 (26–42)	35.5 (25.8-46.5)	33 (26.3-38.8)	0.259
Age groups, years, n (%)				
33	40 (46.5)	22 (47.8)	18 (45.0)	reference
33-42	26 (30.2)	9 (19.6)	17 (42.5)	0.105
> 42	20 (23.3)	15 (32.6)	5 (12.5)	0.133
Clinical signs				
Haemoptysis, n (%)	19 (22.1)	6 (13)	13 (32.5)	0.030
Dyspnoea, n (%)	24 (27.9)	11 (23.9)	13 (32.5)	0.375
Fever, n (%)	74 (86)	41 (89.1)	33 (82.5)	0.376
Weight loss, n (%)	80 (93)	43 (93.5)	37 (92.5)	0.859
Median BMI, kg/m^2^ (25^th^-75^th^ percentiles)	19.3 (17.4-20.8)	18.9 (17.2-21.0)	19.4 (17.5-20.3)	0.698
BMI strata, kg/m^2^				
< 18.5	18 (20.9)	11 (23.9)	7 (17.5)	0.466
≥ 18.5	35 (76.1)	35 (76.1)	33 (82.5)	
BCG vaccination, n (%)	63 (73.3)	33 (71.7)	30 (75.0)	0.733
Diabetes mellitus, n (%)	2 (2.3)	2 (4.3)	0 (0)	0.182
Habits				
Smoking, n (%)	22 (25.6)	17 (37)	5 (12.5)	0.010
Alcohol consumption, n (%)	22 (25.6)	13 (28.3)	9 (22.5)	0.541
Median symptoms duration, weeks (25^th^-75^th^ percentiles)	13 (8–20.3)	12 (6–21)	13 (8–20)	0.744
HIV Positive, n (%)	24 (27.9)	11 (23.9)	13 (32.5)	0.376
Median CD4, /mm^3^ (25^th^-75^th^ percentiles)	187 (151.3-216.3)	187 (151–240)	187 (144–210)	0.697
CD4 strata, /mm^3^				
< 50	3/24 (12.5)	1/11 (9.1)	2/13 (15.4)	1.000
50-200	15/24 (62.5)	7/11 (63.6)	8/13 (61.5)	1.000
> 200	6/24 (25.0)	3/11 (27.3)	3/13 (23.1)	Reference
Chest X-ray				
Number of affected areas > 4, n (%)	46 (53.5)	28 (60.9)	18 (45.0)	0.141
Presence of cavitations, n (%)	60 (69.8)	31 (67.4)	29 (72.5)	0.607

C+, positive culture for *Mycobacterium tuberculosis*; C– , negative culture for *Mycobacterium tuberculosis;* BMI, body mass index.

### Clinical and radiological profiles

Table 1 compares the clinical and radiological characteristics of patients with positive sputum smears at the end of the two month intensive phase of treatment by culture status for *M. tuberculosis*. The duration of symptoms prior to starting treatment was similar between patients with positive culture (C+) and negative cultures for *M. tuberculosis* (C–). The prevalence of selected characteristics of culture positive patients (C+ ) as compared to culture negative patients (C–) were 23.9% vs 32.5% (p = 0.376) for HIV infection, 13% vs. 32.5% (p = 0.03) for haemoptysis and 67.4% vs. 72.5% (p = 0.607) for cavitation on chest X-ray. In a multivariable logistic regression model, the absence of haemoptysis [odd ratio 4.65 (95% confidence interval: 1.14-18.95)] and current smoking [7.26 (1.59-33.23)] were the main determinants of non-conversion of sputum culture after two months of intensive treatment.

### Bacteriological profile

The bacteriological profile of patients is described in Table 2. The prevalence of high bacillary load (3+) at baseline and after two months of intensive treatment was similar in the two groups of patients. Of the 46C + patients, 5 (10.9%) were resistant to isoniazid, 2 (4.3%) were resistant to rifampicin and 2 (4.3%) had multiresistant strains of *M. tuberculosis.*

**Table 2:** **Bacteriological profile of patients with positive sputum smear at the end of the second month of intensive treatment, with positive (C+) or negative (C–) culture for *Mycobacterium tuberculosis***

**Characteristics**	**Overall (n = 86)**	C + (n = 46)*	C– (n = 40)*	**p**
Initial bacillary load > 2+	69 (80.2)	37 (80.4)	32 (80.0)	0.959
Bacillary load at 2-month of treatment > 2+	12 (14.0)	7 (15.2)	5 (12.5)	0.717
Multidrug resistant strain	/	2 (4.3)	/	/
Resistance to rifampicin	/	2 (4.3)	/	/
Resistance to isoniazid	/	5 (10.9)	/	/
Resistance to ethambutol	/	1 (2.3)	/	/
Resistance to streptomycin	/	5 (10.9)	/	/
Resistance to at least one anti-tuberculosis drugs		7 (15.2)	/	/

*7C + patients and 1C- patient were transferred to another centre.

### Outcomes

There was a trend toward lower therapeutic success among C + patients compared with C– ones, although the difference did not reach statistical significance (64.1% vs. 79.5%, p = 0.131). There was a significantly high treatment failure rate among C + patients as compared to C– patients (28.2% vs. 7.7%, p = 0.023; Table 3). The sensitivity and specificity were 78.6% and 55.4% for culture non-conversion after intensive treatment phase, in predicting anti-TB treatment failure. Of the five patients for whom the involved *M. tuberculosis* strain was resistant to at least one of the drugs of the category I regimen, 3 (60%) had treatment failure against 8 (19.5%) among those with non-resistant mycobacterium strains (p = 0.080). The two patients with multiresistant strains had treatment failure.

**Table 3:** **Outcome of patients with positive sputum smear at the end of the second month of intensive treatment, with positive (C+) or negative (C–) culture for *Mycobacterium tuberculosis***

Outcome*	**Overall (n = 78)**	**C + (n = 39)**	**C– (n = 39)**	**p**
Therapeutic success	56 (71.8)	25 (64.1)	31 (79.5)	0.131
Failure	14 (17.9)	11 (28.2)	3 (7.7)	0.023
Loss to follow-up	7 (9.0)	3 (7.7)	4 (10.3)	1.000
Deceased	1 (1.3)	0 (0)	1 (2.6)	1.000

* 7C + patients and 1C- patient were transferred to another centre.

## Discussion

Four main findings emerge from this study conducted in a country with medium endemicity for tuberculosis among patients with positive smear after the intensive phase of treatment for a first episode of SPPTB: 1) over half of the patients with positive smears after the intensive phase of treatment have positive cultures for *M. tuberculosis* (culture non-conversion); 2) current active smoking and absence of haemoptysis are the main determinants of culture non-conversion; 3) about 4% of patients with culture non-conversion have multidrug resistant TB; and 4) treatment failure is very high among patients with positive cultures compared to those with negative cultures.

Our findings suggest that over half of the patients with persistent positive smears on direct examination after the intensive phase of anti-TB treatment also have positive cultures, and therefore remain contagious. In a cohort followed by Su and co-workers [7], Involving 113 patients with positive smears after receiving intensive treatment, 56% also had positive cultures for *M. tuberculosis*. Our findings are also similar to those reported by Ramarokoto et al. in Madagascar [10]. WHO guidelines are currently against extending the intensive phase of treatment among patients who remain smear positive after two months of an intensive treatment with a regimen containing rifampicin [3]. This recommendation essentially is based on the fact that positive sputum smears on direct examination following the intensive phase of anti-TB treatment is not a good indicator of the treatment outcome. It is instead recommended that sputum culture and drug susceptibility tests be conducted in all patients with positive smears at the completion of the intensive treatment and at the end of the 3^rd^ month of treatment. In resource limited countries however, the high incidence of tuberculosis and insufficient equipment remain deterrents to systematic sputum culture and susceptibility tests.

In Cameroon where about 6-13% of patients with smear positive tuberculosis are still positive after completing the intensive phase of treatment [6,11], our findings suggest that per year, about 1200 patients with tuberculosis in the country remain contagious after completing this phase of treatment. The current approach of the National Tuberculosis Control Program(NTCP), which consists in extending this phase by an additional month [2] needs to be clarified by further studies, since despite the extension of the intensive phase, the failure rate is fairly high especially in the subgroup of patients with positive culture. Such a strategy could aid in reducing the spread of the disease by a number of these patients. It is however known that the median time from treatment start to achieving negative smear is about 17.5 days [8]. Therefore, patients with persistent positive smears after two months of intensive treatment are likely a subgroup of patients for whom specific actions are needed to optimise the treatment and care. The adoption therefore in developing countries of modern rapid methods for identifying and testing the susceptibility of *M. tuberculosis* to at least rifampicin should be considered and encouraged. These will help considerably in improving clinical decision making. Even in the absence of multiple drugs resistant tuberculosis, treatment failure was high in group C + patients. These were essentially clinical failure, likely due to poor adherence; suggesting the need for an ongoing supervision of these patients throughout the entire course of their treatment.

None of the baseline radiographic or bacteriologic features was associated with culture non-conversion after intensive treatment among our patients. A study in Taiwan reported the presence of cavitation or condensation on chest X-ray, as well as non-use of Direct Observed Treatment strategy, to be associated with culture non-conversion [7]. Elsewhere, high baseline bacillary load (≥2+) has been associated with delayed culture conversion [4]. In this study, haemoptysis was observed to be significantly associated with patients having culture conversions (C+). The exact reason for this association is not known. In agreement with the results of our study, current smoking at baseline has also been observed to be associated with culture non-conversion in similar studies from Brazil [12] and Spain [13]. It could be speculated that the impairment of local pulmonary defences caused by the persistent irritation of tobacco smoke may play a role. Screening for these factors in settings where culture of mycobacterium are not available, could aid the identification of a subgroup of patients who require more intensive monitoring.

In this study, the prevalence of multidrug resistant *M. tuberculosis* strains in patients with positive cultures was 4.3%. This prevalence is relatively high as compared to rates (0 to 2.7%) reported for patients with a first episode of SPPTB in the same region [14]. This suggests that patients with persistent culture positivity at the end of the 2 month intensive phase of anti-tuberculosis treatment constitute a high risk group for multidrug resistant tuberculosis MDR-TB. As such this group of patients should like retreatment cases be included in the high risk group of patients for MDR-TB so that they could benefit from sputum cultures for identification and drug susceptibility testing of their *M. tuberculosis* strains. This will allow for a timely adjustment of their treatment as opposed to waiting until 3 months as recommended presently by WHO [3]. Cost-effectiveness studies are needed to guide the choice of the appropriate strategies particularly in resources-limited settings.

The present study has some limitations. No sputum smear control examination was done at the end of the extended intensive phase. This would have allowed us to assess the effects of non-conversion after 3 months of intensive treatment on the outcomes of care. Furthermore, no culture was performed at the end of the entire treatment cycle, and the outcome of care was essentially guided by the WHO recommendations, based on smear examination at five or six months of treatment [2,3]. The study also has some major advantages including its prospective nature, the enrolment of both HIV + and HIV- patients, and the relatively large number of patients with persistent positive smears after receiving intensive phase treatment.

## Conclusions

Over half of the patients with a first episode of SPPTB presenting persistent positive smears after an intensive phase of anti-TB treatment have positive cultures for *Mycobacterium tuberculosis* in Yaounde. Treatment failure is more frequent among these patients, even in the absence of MDR-TB. Our findings support the need for a monitoring of the entire treatment cycle in these patients, and the adoption of rapid drug susceptibility tests for patients with persistent positive smears after intensive phase treatment. This will allow timely adjustment of the treatment as opposed to waiting until the end of the third month of treatment as currently done.

## Abbreviations

AFB: Acid-fast bacilli; C–: Negative culture for *M. tuberculosis*; C+: Positive culture for *M. tuberculosis*; DOT: Directly observed treatment; E: Ethambutol; H: Isoniazid; HIV: Human immunodeficiency virus; M. tuberculosis: Mycobacterium tuberculosis; MDR-TB: Multidrug resistant tuberculosis; NTCP: National Tuberculosis Control Program; R: Rifampicin; SPPTB: Smear positive pulmonary tuberculosis; TB: Tuberculosis; WHO: World Health Organisation; YJH: Yaounde Jamot Hospital; Z: Pyrazinamide.

## Competing interests

The authors declare that they have no competing interests in relation with this manuscript.

## Authors’ contribution

EWPY conceived the study, collected data, co-analysed the data and drafted of the manuscript; APK contributed to data analysis, drafting and critical revision of the manuscript; CK supervised the data collection and critically revised the manuscript. All the authors approved the submitted manuscript.

## Pre-publication history

The pre-publication history for this paper can be accessed here:

http://www.biomedcentral.com/1471-2334/14/138/prepub
